# Supplementary material for: The Legionella collagen-like protein employs a distinct binding mechanism for the recognition of host glycosaminoglycans
Source: Nat Commun. 2024 Jun 8;15:4912. doi: 10.1038/s41467-024-49255-4 (PMC11162425; doi:10.1038/s41467-024-49255-4)
Supplement: Supplementary file 1 — Supplementary Information [file 41467_2024_49255_MOESM1_ESM.pdf]

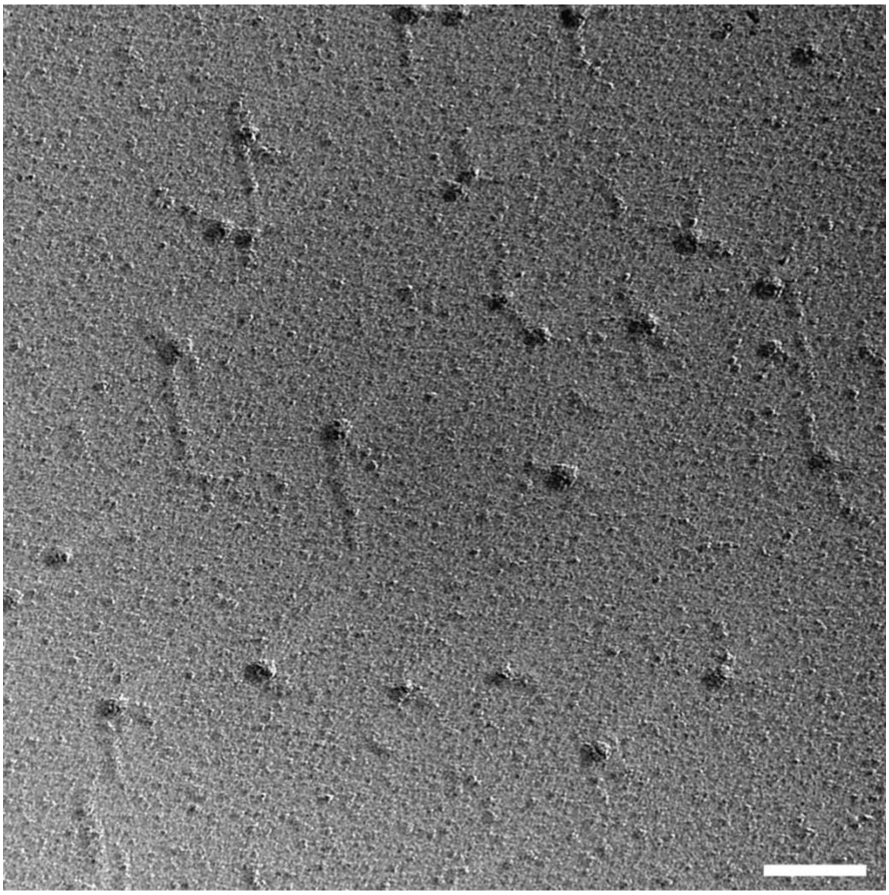

Supplementary Figure 1. Rotary shadowing electron microscopy of Lcl. Micrograph showing lollipop-shaped structures of Lcl trimers. The globular shapes correspond to trimeric C-terminal domains (CTD), while the stalks contain a trimeric collagen triple-helix (CLR). Some globular heads are missing the stalk region due to proteolysis. Variation in stalk lengths is due to differences in the platinum film thickness. The concentration of Lcl was 5 µg/ml. Scale bar: 50 nm.

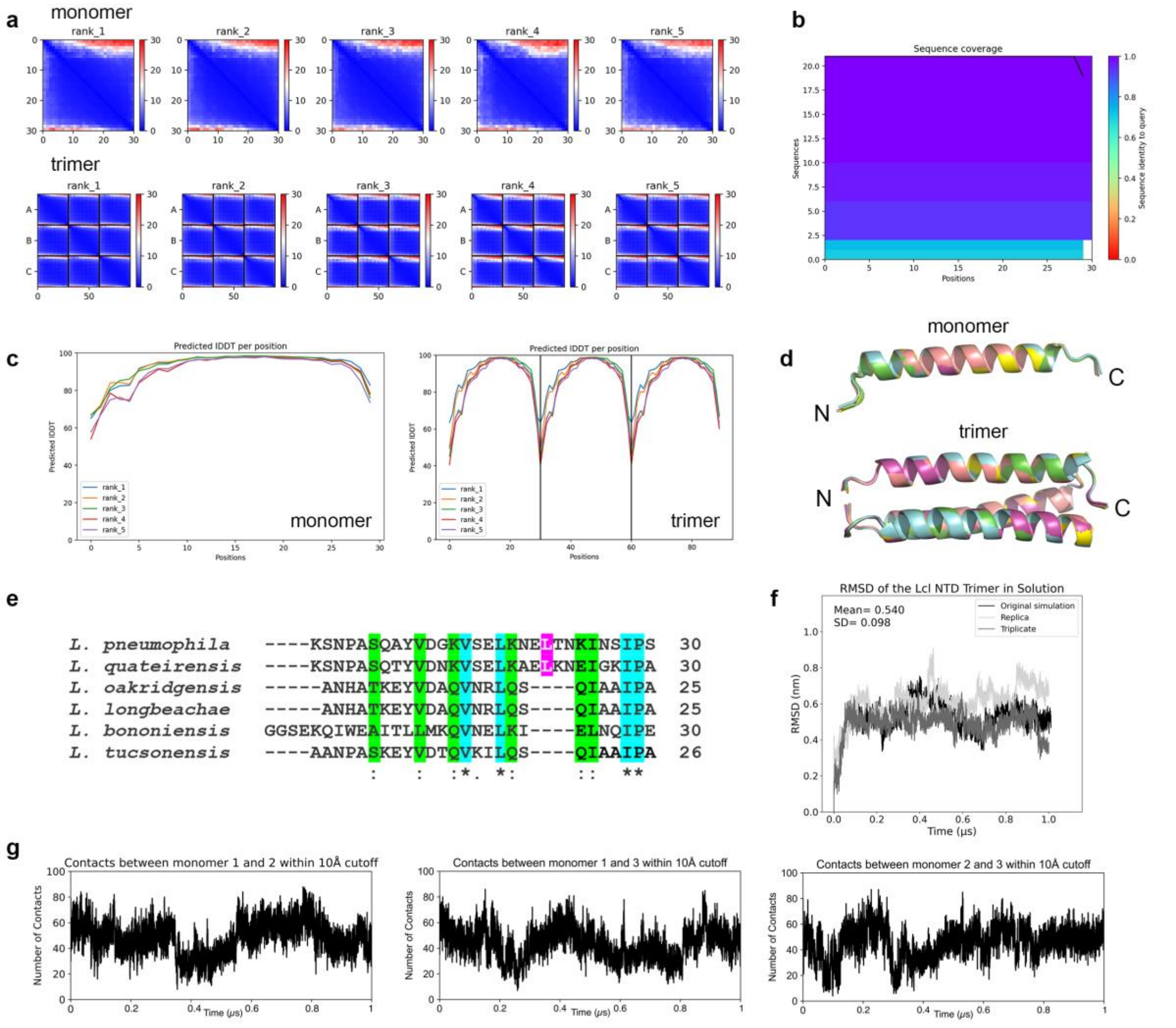

Supplementary Figure 2. AlphaFold2 modelling and analysis of monomeric and trimeric Lcl-N. **a** Predicted Aligned Error (PAE) analysis of the five top ranking models for both monomer and trimer. Low PAE values throughout the matrix indicates a well folded single domain. **b** Sequence coverage and identity used during modelling. Only monomer is shown. **c** Local Distance Difference Test (lDDT) analysis against residue position for the five top ranking models for both monomer and trimer. High lDDT values (<90) indicate a high accuracy in atomic positions for that residue. Lower values (<70) indicate low confidence atomic positioning, which is observed in dynamic regions. **d** Superposed top five ranked monomeric and trimeric models with the termini highlighted (N/C: N-/C-terminus). **e** Sequence alignment of the Lcl-N region across the *Legionella* genus. Only sequences are shown from species that contain a periplasm signal sequence in Lcl. Amino acid positions with 100% identical, >50% identical and similar residues are indicated by asterisk (*; cyan), colon (:; green), and period (.), respectively. An insertion sequence is seen in *L. pneumophila* and *L. quateirensis*, resulting in an additional helical turn, and containing a conserved leucine (purple). **f** MD analysis of the Lcl-N trimer showing backbone RMSD over the course of three independent 1 µs MD simulations. **g** Contacts between the monomeric NTD helices detecting during the MD simulation. The number of contacts between the C_α atoms from one peptide versus another using a 10 Å cut-off, for all 3 chains, respectively. The helices align closely in a head-head conformation, although monomers 2 and 3 are not as closely attracted as monomers 1 to 2 and 1 to 3. Source data for the molecular dynamics data (**e, f**) are available at https://zenodo.org/doi/10.5281/zenodo.10961237.

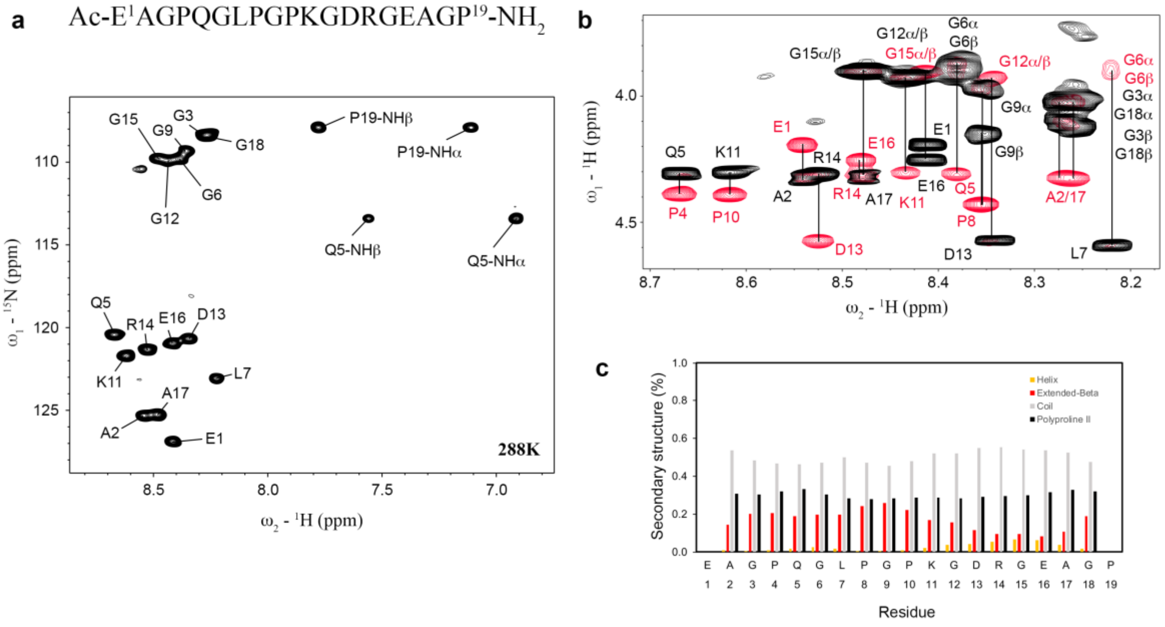

Supplementary Figure 3. Solution NMR spectroscopy analyses of unlabelled CLR peptide recorded at 288 K. a ^{1}H–^{15}N SOFAST-HMQC spectrum of the CLR peptide with amide resonance assignments shown. Peptide sequence is shown above. **b** ^{1}H–^{1}H TOCSY (black) and ^{1}H–^{1}H ROESY (red) spectra expanded on the NH-H_α region. A typical pattern of polyproline II structure is seen with strong NOE correlation observed between NH (i) and H_α (i-1). **c** Secondary structure propensity of monomeric CLR peptide derived from backbone chemical shifts (C_α, C_β, H_α, N, NH) calculated using δ2D[1].

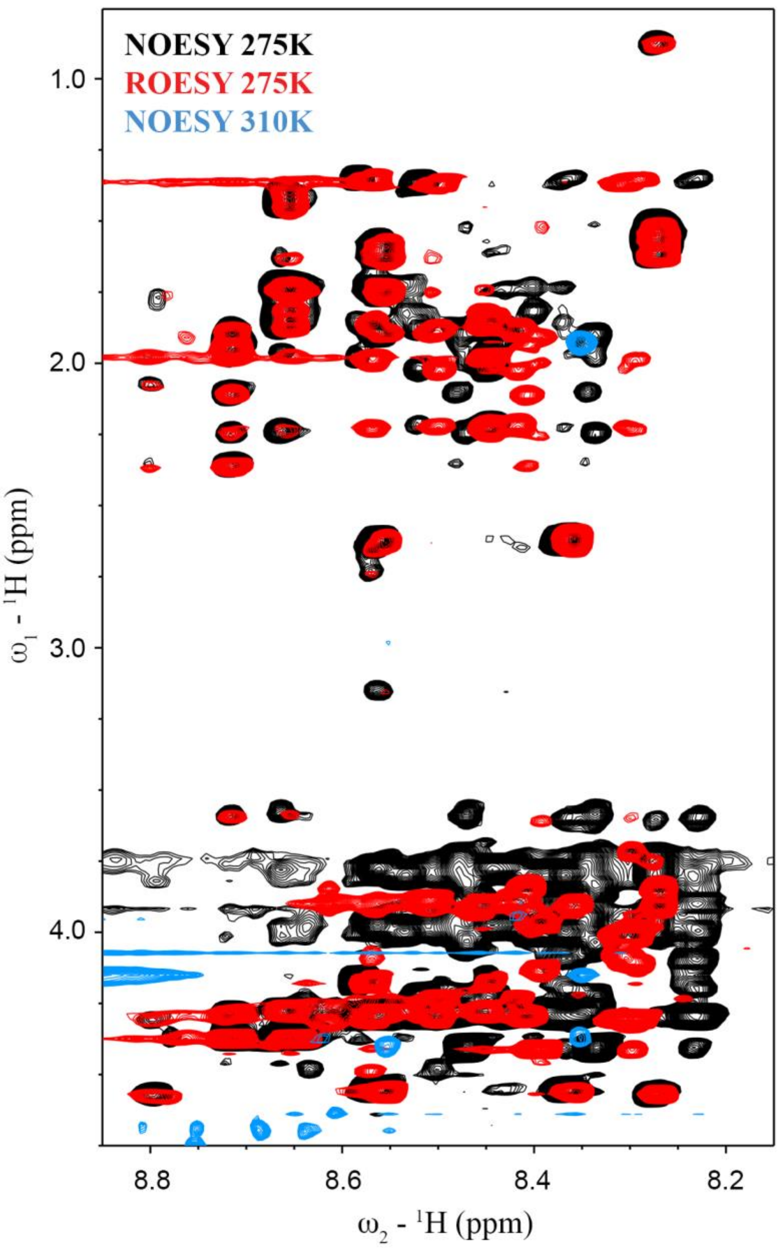

Supplementary Figure 4.| Solution NMR spectroscopy analyses of $^{13}C^{15}N$ glycine labelled CLR peptide. ^{1}H–^{1}H NOESY spectra (mixing time 240 ms) recorded at 275 K (black) and 310 K (blue), and ^{1}H–^{1}H ROESY (mixing time 200 ms) spectrum recorded at 275 K (red) expanded on the amide region. Significant differences are observed between the NOESY and ROESY spectra at 275 K and significant spectral broadening is observed in the NOESY spectrum at 310 K. This indicates that the peptide is in equilibrium between monomeric and trimeric states.

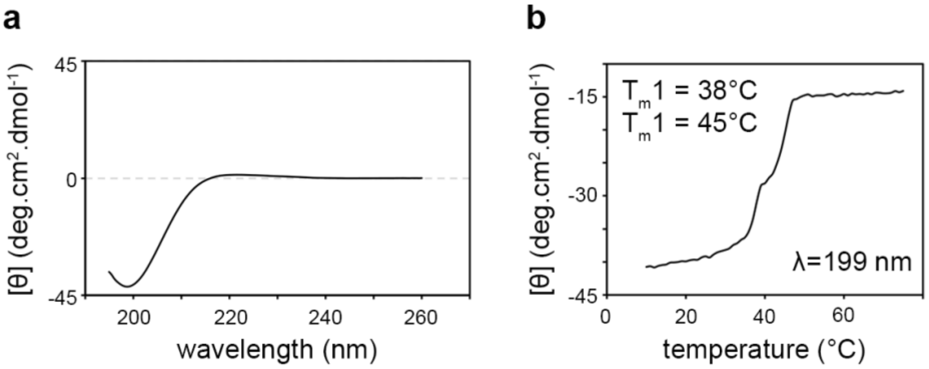

Supplementary Figure 5. Circular dichroism (CD) spectra of Lcl. a CD spectra of Lcl recorded at 10°C with negative peak at 199 nm and maximum peak at 222 nm. **b** Thermal denaturation of Lcl recorded between 10°C and 75°C at 199 nm. Two melting temperatures were determined at 38°C and 45°C. Source data are provided as an accompanying Source Data file.

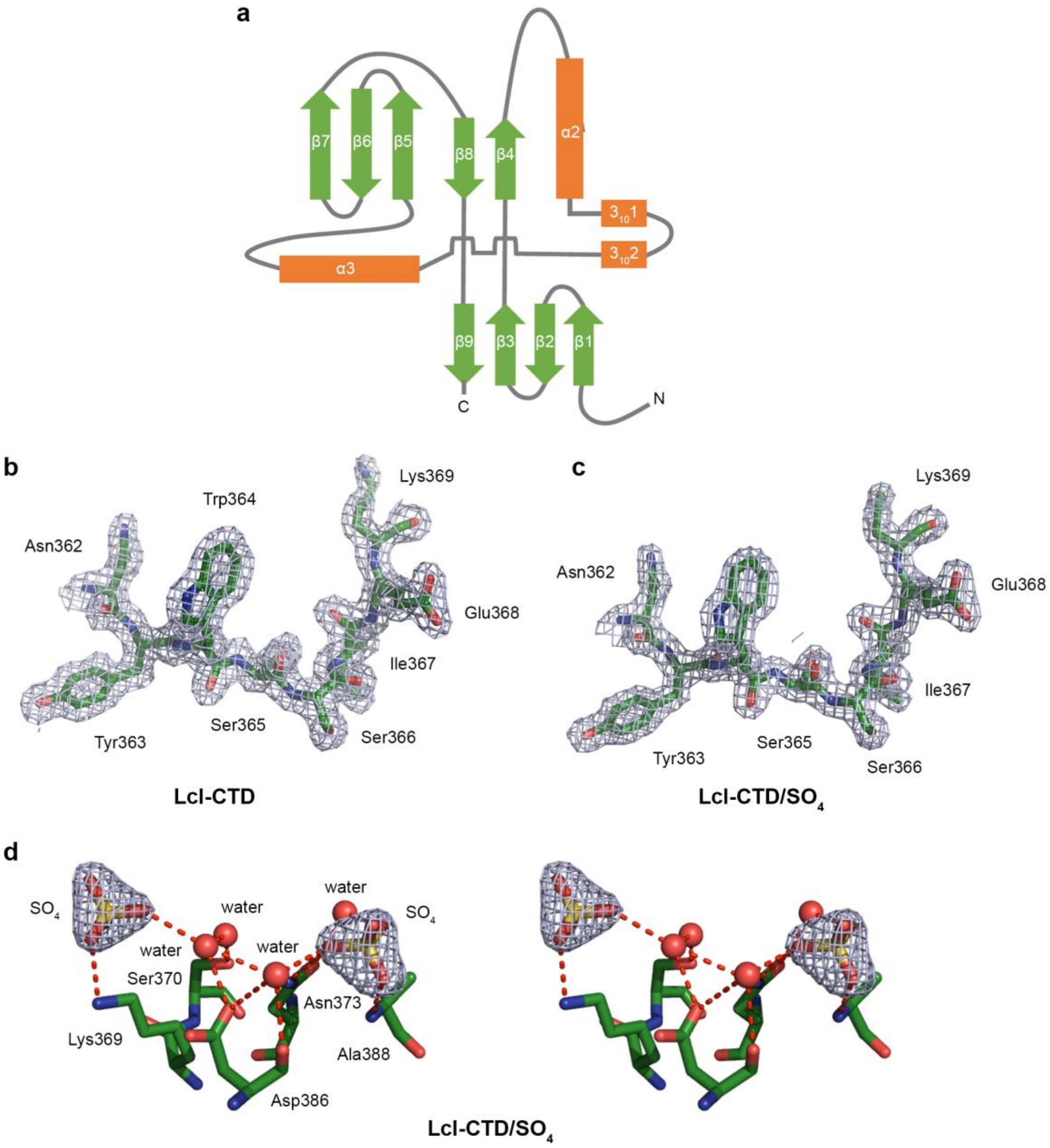

Supplementary Figure 6. Structures of Lcl-CTD and Lcl-CTD/SO_4. a Topology diagram showing an individual Lcl-CTD domain. α-helix and 3_{10}-helix secondary structure are coloured orange, β-sheet secondary structure is coloured green, and loops are grey. Helices are labelled 1-2 and β-sheets are labelled 1-9. **b,c** An example of the electron-density quality of the Lcl-CTD and Lcl-CTD/SO_4 structures. Residues Asn362 to Lys369 of chain A are shown as sticks with a σA-weighted 2Fo – Fc map contoured at 1.5 rms electron density. **d** Cross-eye stereo view of an omit map contoured at 1.5 rms electron density, for the two bound SO_4^{2-} ions in the Lcl-CTD/SO_4 structure. Direct and indirect hydrogen bonding (red dashed line) is observed between the SO_4^{2-} ions and Lys369, Ser370, Asn373, Asp386 and Ala388 in chain A (sticks).

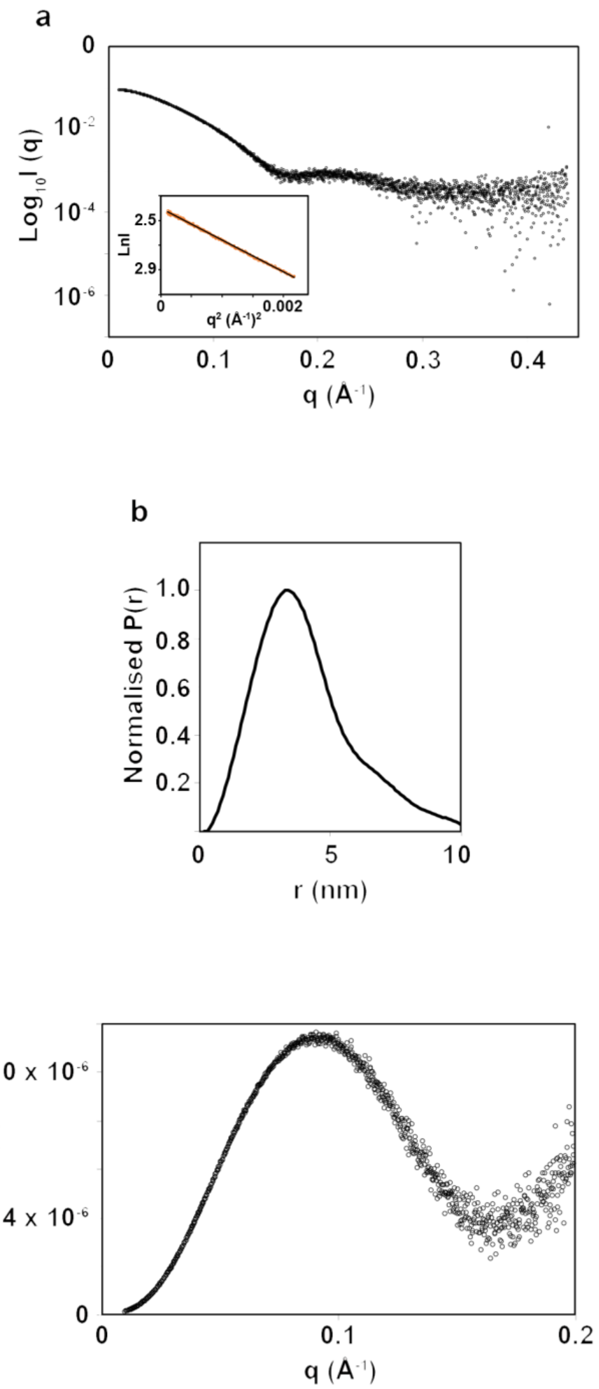

Supplementary Figure 7. SAXS analysis of Lcl-CTD. a Experimental scattering curve of Lcl-CTD (black open circles). Inset: Guinier region (orange open circles) and linear regression (black line) for R_g evaluation. **b** Shape distribution [P(r)] function derived from SAXS analysis for Lcl-CTD. **c** Kratky plot indicates that Lcl-CTD has dynamic properties in solution.

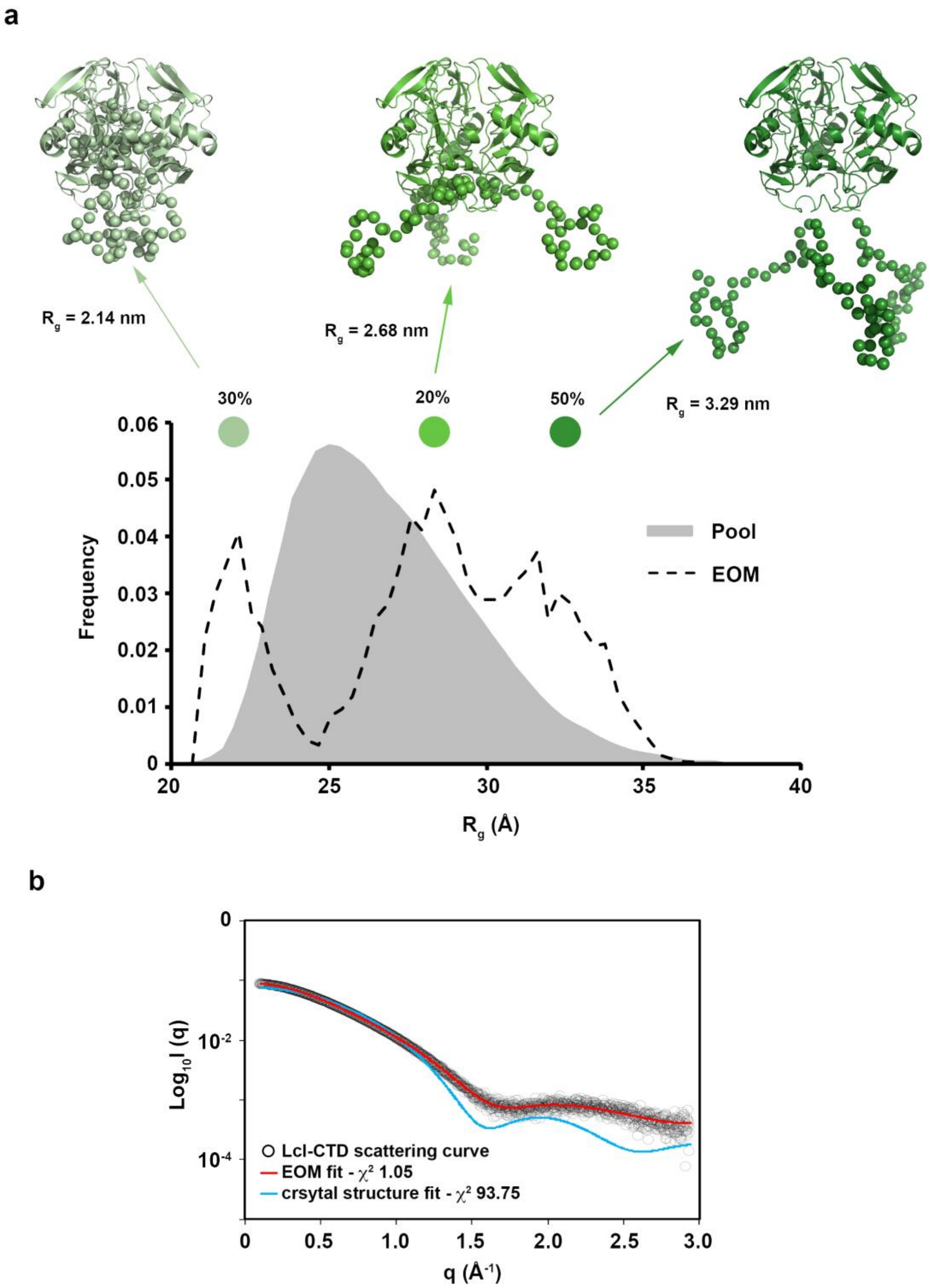

Supplementary Figure 8. SAXS derived model of Lcl-CTD in solution. **a** EOM yielded three distinct populations: extended N-terminus (50%), partially extended N-terminus (20%) and compact N-terminus (30%). Lcl-CTD models corresponding to the centre of each population are shown. **b** EOM (red line) and crystal structure (blue) fit to the Lcl-CTD SAXS data (black open circles) with χ^2 of 1.05 and 93.75, respectively. Source data are provided as an accompanying Source Data file.

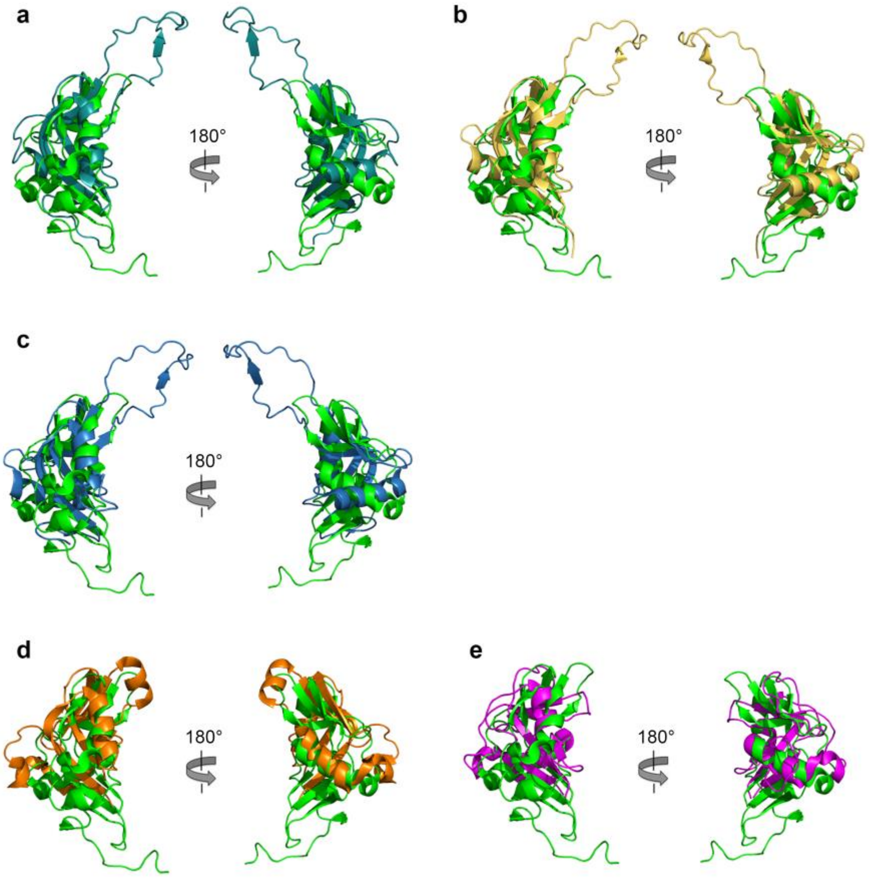

Supplementary Figure 9. Structural comparison of the Lcl-CTD monomer with other C-type lectin-like domains. Homologous structures identified using the Dali server with Z-scores >8.0 are shown[2]. **a** Cartoon representation of Lcl-CTD (green) superimposed on the platelet glycoprotein Ib-α binding protein mucrocetin from the venom of *Trimeresurus mucrosquamatus* (teal; Protein Data Bank (PDB) ID code 1v4l[3]; chain B residues 201–325; Z-score 9.7; root mean squared deviated (rmsd) 2.7 Å). **b** Cartoon representation of Lcl-CTD (green) superimposed on the integrin binding protein EMS16 from the venom of *Echis multisquamatus* (yellow; PDB ID code 1v7p[4]; chain A residues 1–127; Z-score 9.1; rmsd 2.7 Å). **c** Cartoon representation of Lcl-CTD (green) superimposed on the integrin binding protein rhodocetin from the venom of *Calloselasma rhodostoma* (blue; PDB ID code 6nd8; chain B residues 1–122; Z-score 8.5; rmsd 2.8 Å). **d** Cartoon representation of Lcl-CTD (green) superimposed on the adhesin protein intimin from enteropathogenic *Escherichia coli* (orange; PDB ID code 1f00[5]; chain I residues 840–939; Z-score 8.3; rmsd 3.5 Å). **e** Cartoon representation of Lcl-CTD (green) superimposed on the integrin binding protein invasin from *Yersinia pseudotuberculosis* (purple; PDB ID code 1cwv[6]; chain A residues 887–986; Z-score 8.1; rmsd 2.9 Å).

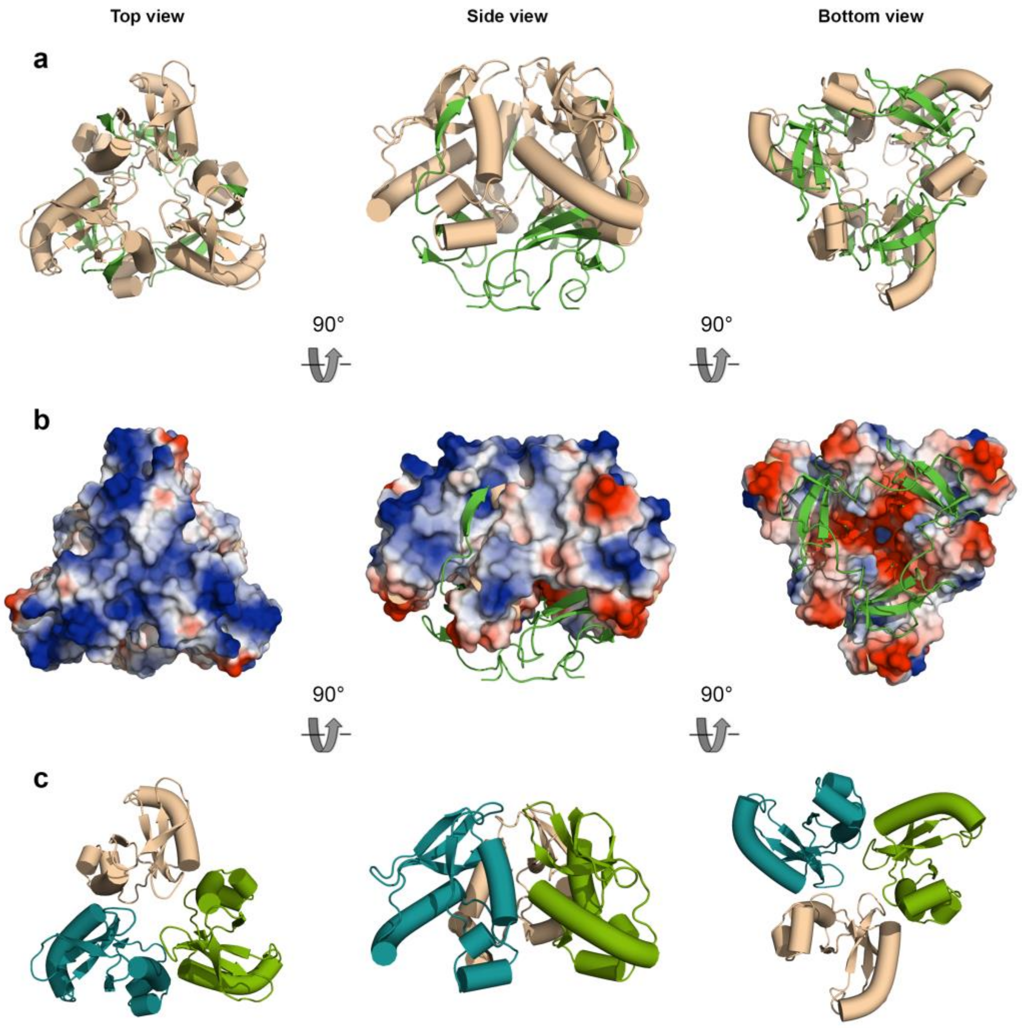

d

```
DUF1566     GTVTDNLTGLMWQRCAEDALAYCAALNAAGFAGYSDWRLPTIRELLSLVD     50
Lcl 130b    -----------WDA--QDSITNPAHFD-NEGKLYSDWRLPTRFELNLIYM     350
                       *:   :*:::  * ::      ********  **   :

DUF1566     PAI-NTTAFPNTYWSSTTSAGNPDYAWAVNFADGSVGYGNKSSGYYVRLVR    100
Lcl 130b    MRNELGNFLAGNYWSSIEKSSA--NSWVFNSKTGEIKDIAKSKTAAVRAVR    399
                    :   ****   :     :*  *   * :    **    ** **
```

Supplementary Figure 10. DUF1566/pfam07603 motif within Lcl-CTD. a Cartoon representation of trimeric Lcl-CTD with the DUF1566 regions coloured wheat, and the non-DUF1566 S1-S4 strands of Lcl-CTD coloured green. **b** Electrostatic surface potential representation of the DUF1566 region within the Lcl-CTD trimer, with the Lcl-CTD S1-S4 strands shown as green cartoon. **c** Cartoon representation of the isolated DUF1566 trimer from Lcl-CTD. **d** Sequence alignment of the consensus DUF1566 motif and the corresponding region within *L. pneumophila* 130b Lcl. Identical residues are highlighted with an asterisk (*), and residues that are similar are highlighted with a colon (:). Completely conserved positions in the DUF1566 sequence that are also identical in Lcl are coloured red, and those that are not identical are coloured blue.

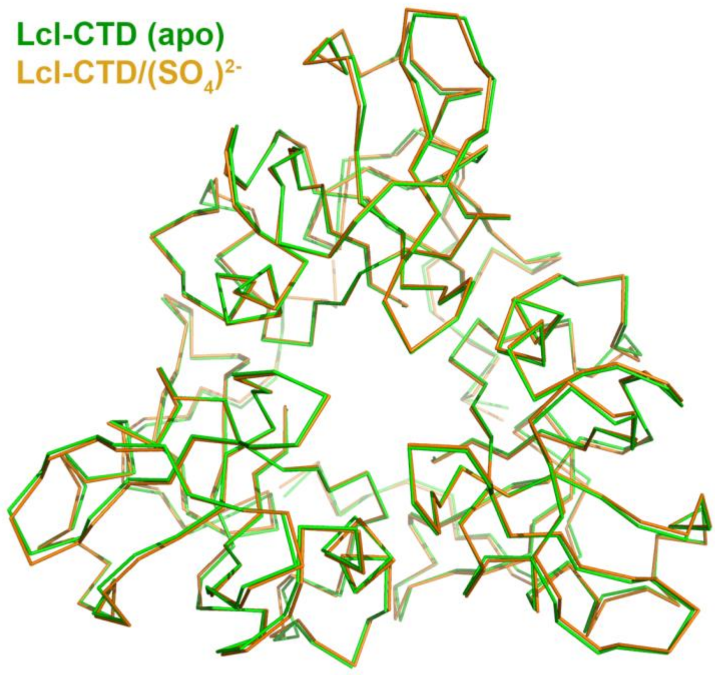

Supplementary Figure 11. Structural comparison of the Lcl-CTD trimer in the presence and absence of sulphate ions. Lcl-CTD trimer backbone structures are shown as ribbons in green (no sulphate) and orange (bound to sulphate ions).

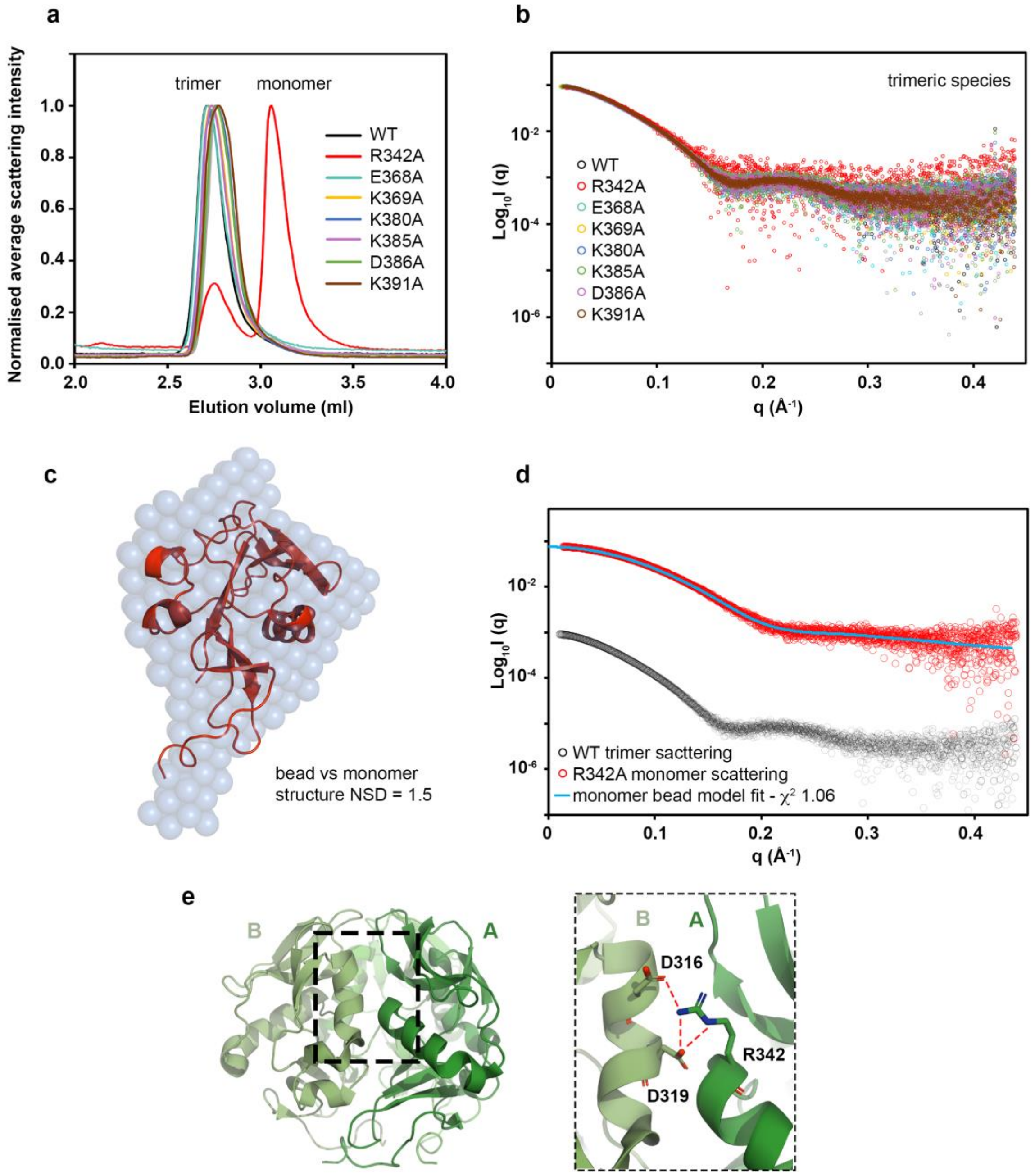

Supplementary Figure 12. SAXS analysis of Lcl-CTD mutants. a Size-exclusion chromatography coupled with SAXS profile of wild-type and engineered Lcl-CTD. **b** Experimental scattering curves of trimeric forms of wild-type and engineered Lcl-CTD. **c** DAMMIF bead model of monomeric Lcl-CTD R342A superimposed with chain A from Lcl-CTD, with normalized spatial discrepancy (NSD) score of 1.5. **d** Monomer Lcl-CTD crystal structure fit to monomeric Lcl-CTD R342A SAXS data (red open circles) with χ^2 of 1.06. Experimental scattering curve of trimeric wild-type Lcl-CTD (black open circles) is shown for comparison. **e** Cartoon of Lcl-CTD trimer highlighting inter-chain hydrogen bonding between Arg342 (chain A) and Asp316 and Asp319 (chain B).

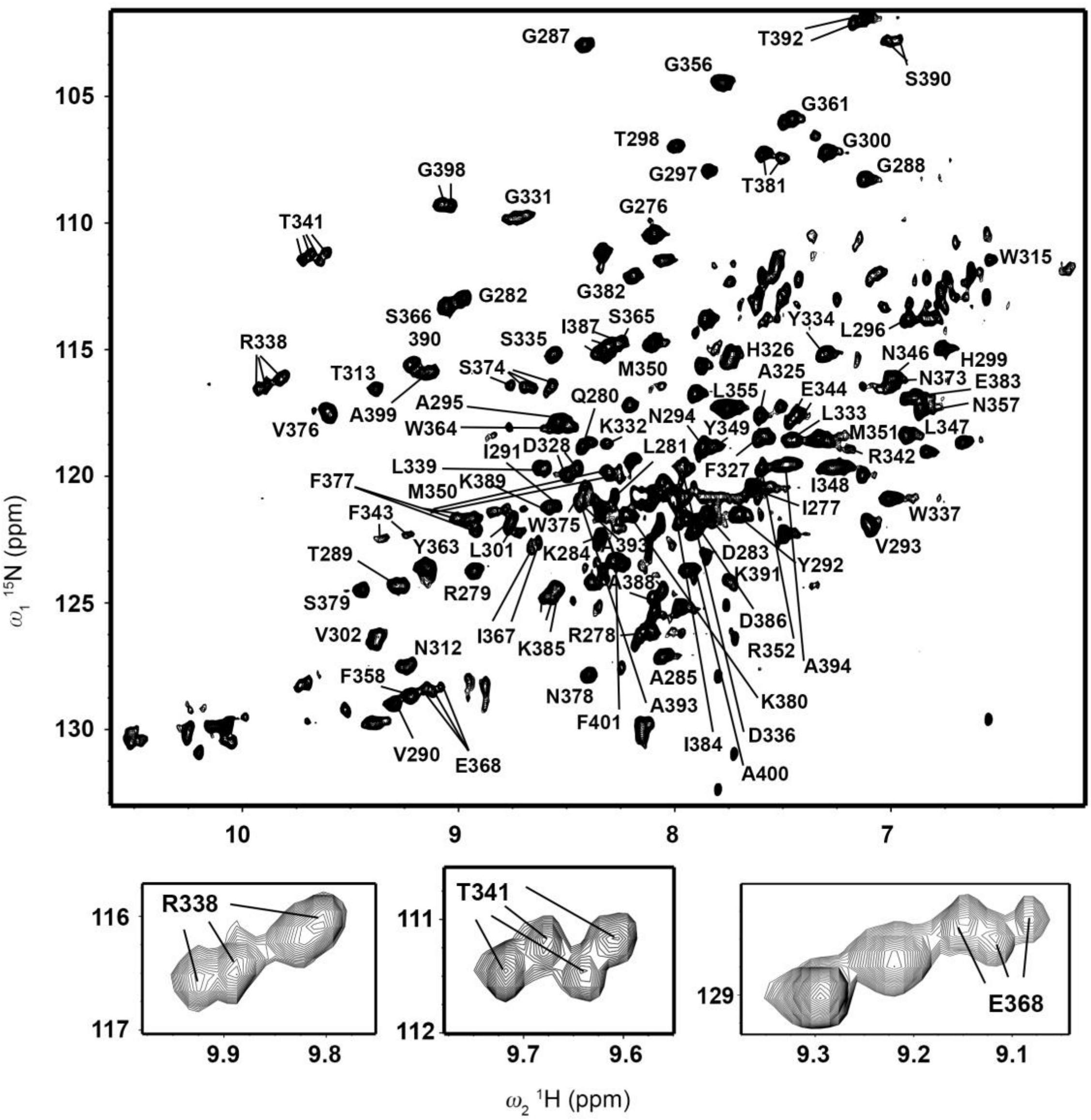

Supplementary Figure 13. Solution NMR spectroscopy of the Lcl C-terminal domain. ^{1}H–^{15}N HSQC TROSY spectrum with backbone amide resonance assignments shown. Boxed out areas are examples of residues that display conformation exchange.

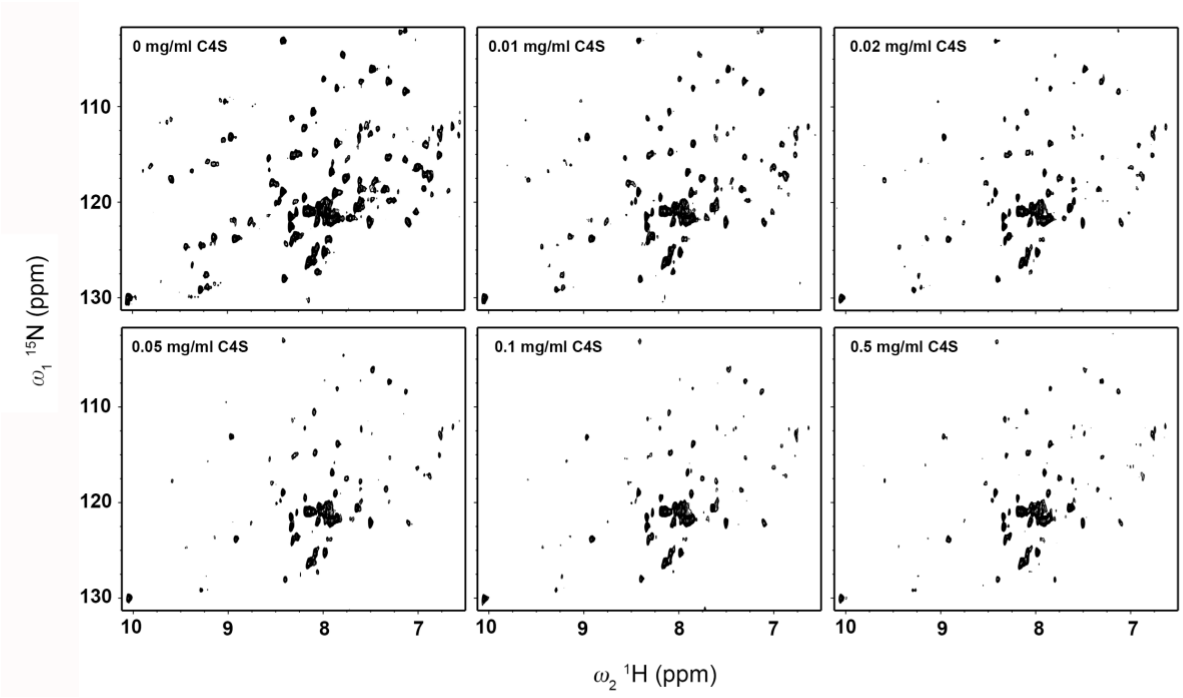

Supplementary Figure 14. NMR titration of C4S against Lcl-CTD. ^{1}H–^{15}N HSQC TROSY spectra of $^{2}H^{15}N$ labelled Lcl-CTD incubated with increasing concentrations of C4S extracted from bovine trachea.

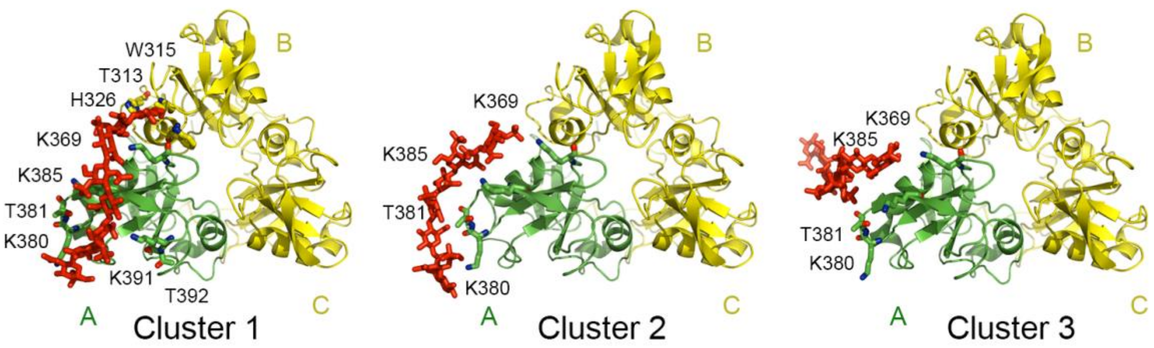

Supplementary Figure 15. Docking of C4S dp8 against monomeric Lcl-CTD. Top three HADDOCK clusters are shown. C4S is shown as sticks and coloured red, while monomeric Lcl-CTD is shown as a green cartoon. Docking was carried out on monomeric Lcl-CTD, but here it is shown superimposed onto the crystal trimer (yellow). The trimeric structure shown in Cluster 1 is equivalent to the HM model used for subsequent MD simulations.

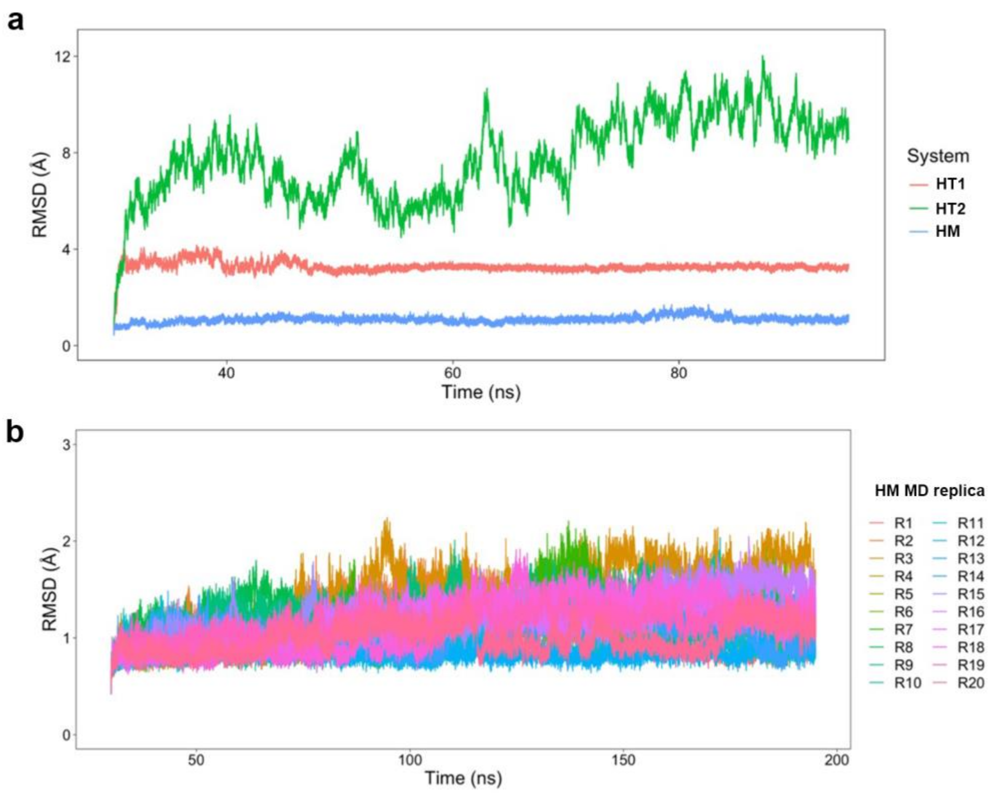

Supplementary Figure 16. Stability of Lcl-CTD/C4S during Molecular Dynamics simulations. a Time evolutions of the RMSD of C_α atoms from the starting structure before energy minimisation for Lcl-CTD in complex with C4S. The HT1 and HT2 plots represent MD simulations starting from different HADDOCK models where one molecule of C4S dp8 was docked against a trimer of Lcl-CTD (3:1 Lcl-CTD:C4S). The HM plot represents a simulation starting from the HADDOCK model used for subsequent analysis. During HADDOCK one molecule of C4S dp8 was docked against a monomer (chain A) of Lcl-CTD (1:1 Lcl-CTD:C4S) and then prior to running the MD simulations it was reconstituted as a trimer (3:1 Lcl-CTD:C4S). **b** The RMSD values for all the replicas starting from the HM model. For **a** and **b**, RMSD values were calculated after best-fit superimposition of each frame to the reference structure and the highly flexible N-terminal residues (271-277) were not included in the calculation. The initial equilibration stages with positional restraints were omitted from the plot. Source data are available at https://zenodo.org/doi/10.5281/zenodo.10974841.

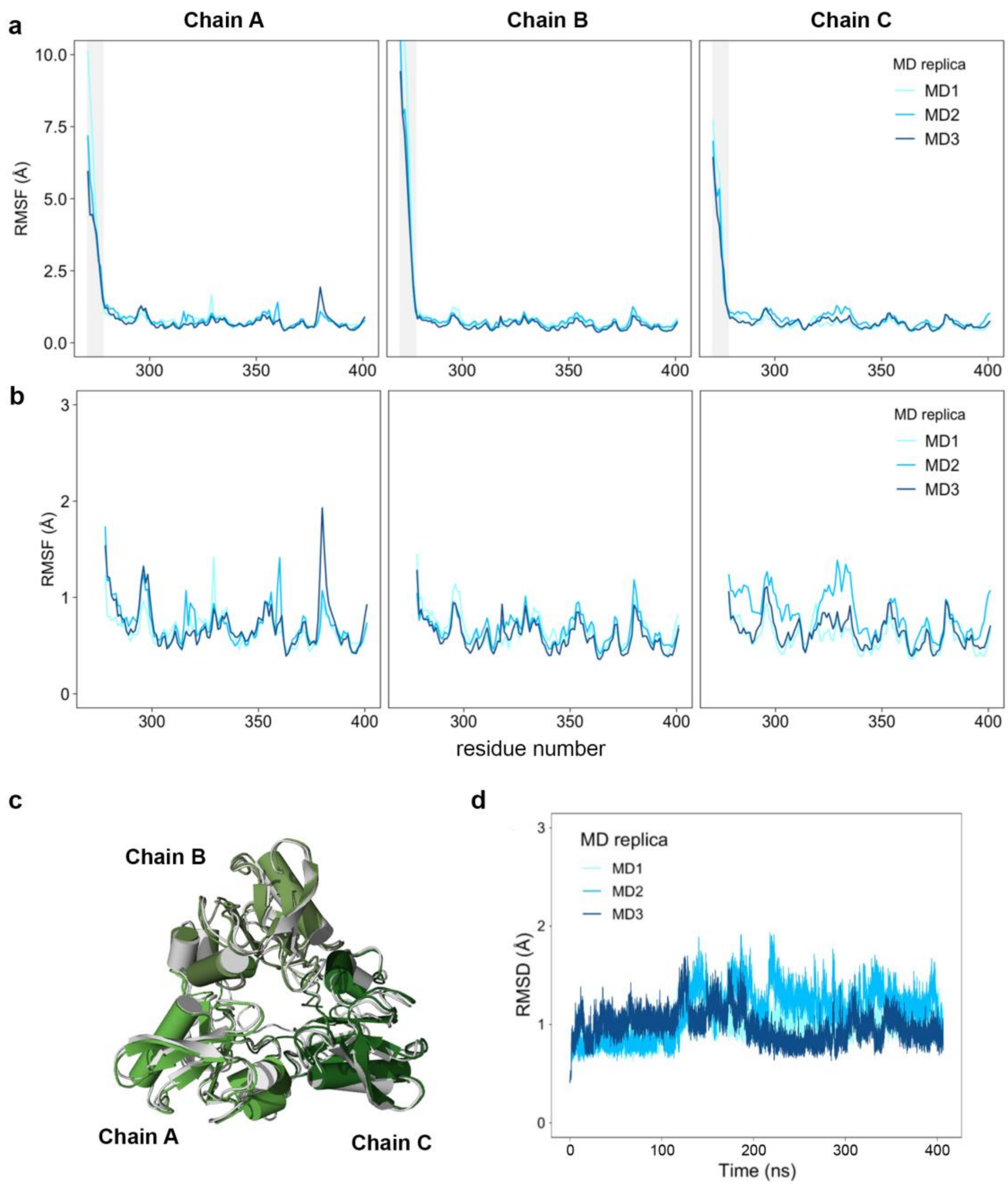

Supplementary Figure 17. Molecular Dynamics simulations of Lcl-CTD. a,b RMSF profiles for each monomer and each replica of the Lcl-CTD simulations calculated with (**a**) and without (**b**) the N-terminal residues (271-277). In **a**, the grey area highlights the position of these residues for each monomer. RMSF values are calculated considering only C_α atoms and on frames saved every 1 ps. **c** Representative structure of the most populated cluster (white, population=79.7%) from the MD simulations superimposed to the initial crystal structure (green hues). **d** Time evolution of the RMSD of C_α atoms from the starting structure before energy minimisation for Lcl-CTD alone. Source data are available at https://zenodo.org/doi/10.5281/zenodo.10974841.

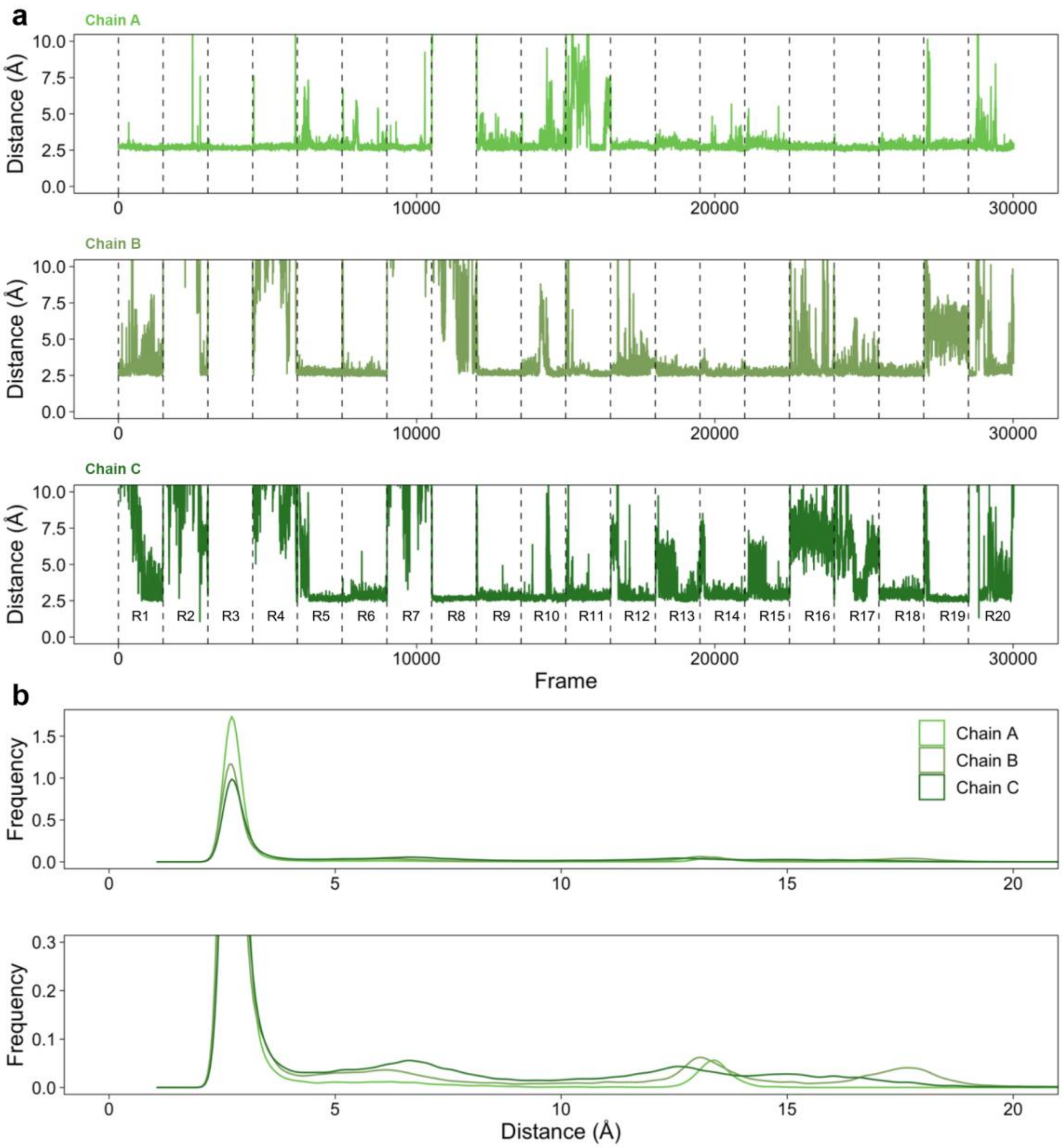

Supplementary Figure 18. C4S binding to Lcl-CTD. a Time evolution of the distance between C4S and each chain of the Lcl-CTD trimer during the 20 MD replicas (production only). A black dotted vertical line separates different replicas. The distance was calculated as the minimum distance between all possible pairs of non-hydrogen atoms from C4S and Lcl-CTD. Analysis of the distances shows that C4S can be in contact with a single chain (36.2% of the frames from all replicas), 2 chains (34.8%) or across the whole trimer (27.7%). **b** Histograms of the distance values shown in (**a**), calculated using all the replicas for each chain. The full range of frequency values is shown in the top panel, while a smaller scale is used for the bottom panel to better show the shape of the curve for larger distance values. Source data are available at https://zenodo.org/doi/10.5281/zenodo.10974841.

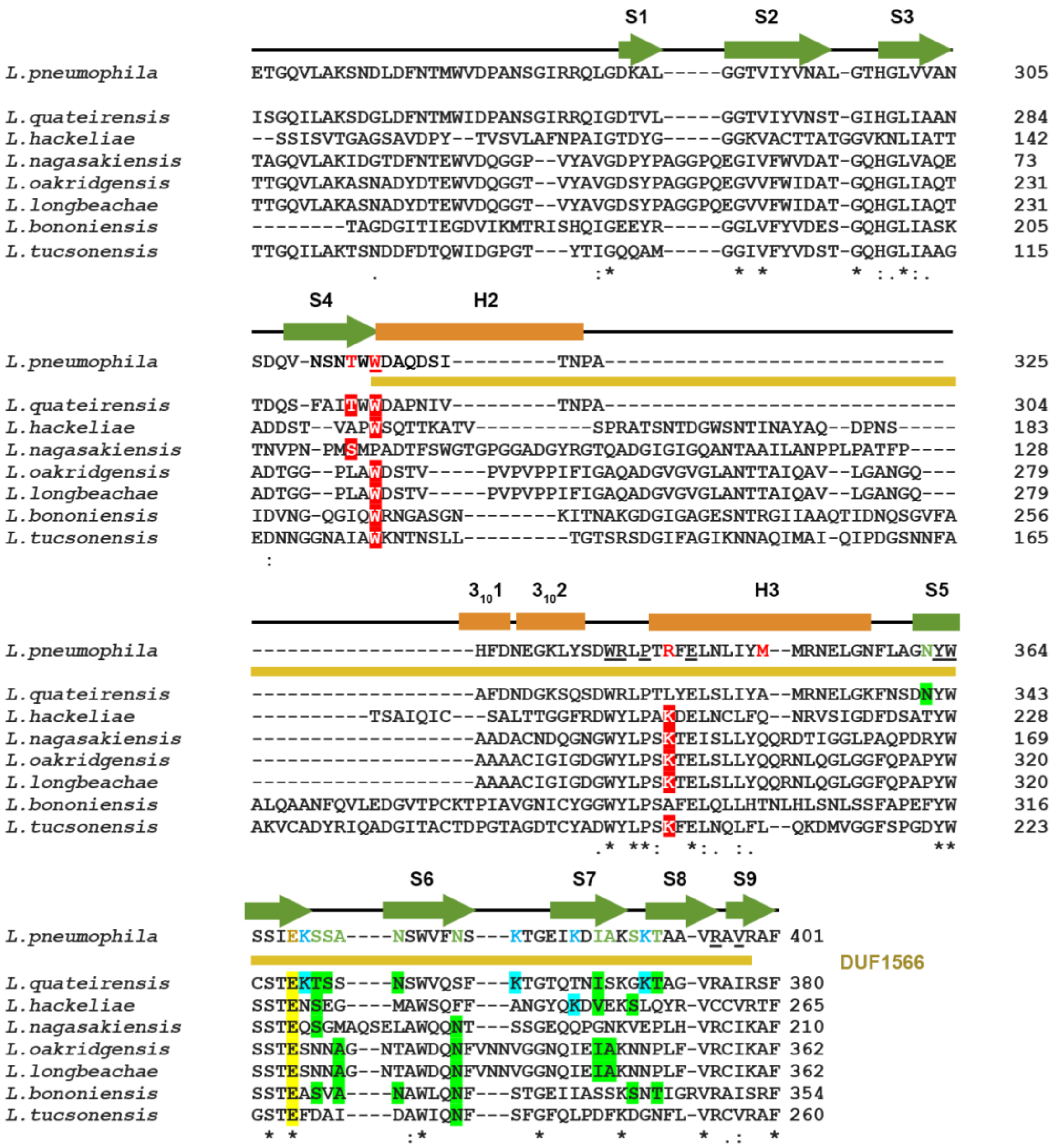

Supplementary Figure 19. Sequence alignment of the Lcl-CTD region across the *Legionella* genus. Amino acid positions with 100% identical, >50% identical and similar residues are indicated by asterisk (*), colon (:), and period (.), respectively. Residues in *L. pneumophila* 130b Lcl-CTD that influence GAG binding are coloured red (identified by NMR), green (identified by MD), yellow (internal glutamate; identified by ELISA) and blue (surface lysines; identified by ELISA). Identical residues in other species are shaded in the same colour. Secondary structure elements and the position of DUF1566 are shown for *L. pneumophila* 130b Lcl-CTD, with related residues in DUF1566 that are highly conserved, underlined in the 130b sequence.

Supplementary Table 1. Domain boundaries of Lcl from *L. pneumophila* 130b strain

The predicted N-terminal amphipathic helix is underlined with residues predicted to form the hydrophobic face in bold[7].

Flexible N-terminus<br>(residues 1 to 30)	KSNPASQAY**V**DGK**V**SE**L**KNE**L**TNK**I**NSIPS
CLR region<br>(residues 31 to 251)	GPQGPRGDKGEAGPKGDRGEAGPQGLPGPKGDRGEAGPQGLPGPKGDRGEAGPQGLPGPKGDRG<br>EAGPQGLPGPKGDRGEAGPQGLPGPQGLPGPKGDKGEAGPQGLPGPKGDKGEAGPQGLPGPKGD<br>KGEAGAVGPQGMPGPKGDKGEAGPQGLPGPKGDRGEAGPQGLPGPKGDRGEAGPQGLPGPKGDK<br>GETGAVGPQGMPGPKGEAGDDGQGVPAGG
C-terminal domain (CTD)<br>(residues 252 to 401)	ETGQVLAKSNDLDFNTMWVDPANSGIRRQLGDKALGGTVIYVNALGTHGLVVANSDQVNSNTWW<br>DAQDSITNPAHFDNEGKLYSDWRLPTRFELNLIYMMRNELGNFLAGNYWSSIEKSSANSWVFNS<br>KTGEIKDIAKSKTAAVRAVRAF

Supplementary Table 2. X-ray data collection, phasing and refinement statistics

	Lcl-CTD	Lcl-CTD/SO_4
**Data collection**		
Space group	C2	P321
Cell dimensions		
a, *b*, *c* (Å)	91.60, 52.87, 97.27	74.91, 74.91, 94.97
α, β, γ (°)	90, 90, 121.09	90, 90, 120
Resolution (Å)	45.56-1.90 (1.95-1.90) *	64.91-1.90 (1.93-1.90) *
R_{merge} (%)	0.058 (0.148)	0.074 (0.541)
$I/\sigma I$	16.7 (6.7)	28.7 (5.8)
Completeness (%)	99.3 (97.3)	100.0 (99.8)
Redundancy	3.3 (2.8)	19.3 (14.8)
**Refinement**		
Resolution (Å)	45.56-1.90 (1.95-1.90) *	64.87-1.90 (1.93-1.90) *
No. reflections	34006	24856
R_{work} / R_{free}	15.8 / 18.8	16.0 / 18.7
No. atoms		
Protein	3087	2058
Ligand/ion	0	10
Water	397	194
B-factors		
Protein	19.1	27.8
Ligand/ion	-	34.5
Water	28.3	35.8
R.m.s. deviations		
Bond lengths (Å)	0.005	0.008
Bond angles (°)	1.180	1.462
**PDB Code**	**8Q4E**	**8QK8**

*Values in parentheses are for highest-resolution shell.
Each structure was derived from one crystal.

Supplementary Table 3. MD system setup

	Lcl-N	Lcl-CTD	Lcl-CTD/C4S
Box dimensions			
d (nm)	6.62	8.68	8.71
No. atoms			
Total	29401	49989	50096
Carbohydrate – C4S	0	0	199
Water	27969	43848	43758
Salt concentration (M)	0.15	0.1	0.1

Supplementary Table 4. SAXS data and refinement statistics

	WT	R342A peak 1	R342A peak 2	E368A	K369A	K380A	K385A	D386A	K391A
**Data collection**									
Instrument	BioSAXS beamline B21 (Diamond Light Source, UK)								
Detector	Pilatus 2M								
Wavelength (Å)	1.0								
q-range (poulations)	0.004-0.44								
Exposure time (s)	3								
Temperature (K)	293								
**Structural parameters and molecular mass determination**									
$I(0)$ (cm^{-1}) (from Guinier)	0.091	0.091	0.075	0.090	0.091	0.094	0.092	0.093	0.093
R_g (nm) (from Guinier)	2.77 ± 0.10	2.68 ± 0.04	1.84 ± 0.15	2.79 ± 0.36	2.78 ± 0.18	2.79 ± 0.04	2.81 ± 0.18	2.83 ± 0.02	2.77 ± 0.01
$I(0)$ (cm^{-1}) (from P(r))	0.091	0.091	0.075	0.090	0.091	0.094	0.092	0.093	0.093
R_g (nm) (from P(r))	2.85 ± 0.01	2.75 ± 0.01	1.85 ± 0.01	2.90 ± 0.01	2.85 ± 0.01	2.86 ± 0.01	2.88 ± 0.01	2.84 ± 0.01	2.83 ± 0.01
D_{max} (nm)	9.70	9.38	6.44	9.87	9.73	9.76	9.84	9.77	9.49
Porod volume (nm^3)	105	79	33	106	106	105	100	108	104
MW (kDa) (from sequence)	18549	18464	18464	18475	18492	18492	18492	18479	18492
MW (kDa) (from SAXS)	50877	47126	18809	50626	51962	51517	51667	51522	51493
**Software**									
Primary data reduction	DAWN processing pipeline								
Data processing and analysis	ATSAS								
Shape/bead modelling	DAMMIF and DAMMIN								
Modelling	MODELLER and EOM2								
**SASDB Code**	**SASD-UG7**	**SASD-UH7**	**SASD-UJ7**	**SASD-UK7**	**SASD-UL7**	**SASD-UM7**	**SASD-UN7**	**SASD-UP7**	**SASD-UQ7**

Supplementary Table 5. SAXS ensemble optimization parameters.

R_{flex} (random) (%)	88.10 (84.55)
R_{sig}	4.40
χ^2	1.05
Cluster 1 (%)	30
Cluster 2 (%)	20
Cluster 3 (%)	50
Final ensemble R_g (nm)	2.82
Final ensemble D_{max} (nm)	9.18

Supplementary Table 6. Average frequency of occurrence of Lcl-CTD/C4S contacts during MD simulations.

**Residue[a]**	**Chain A[b] (%)**	**Chain B[b] (%)**	**Chain C[b] (%)**
S371	40.9	40.3	37.6
A372	29.2	31.1	25.7
**K391**	32.4	24.8	19.3
S390	20.9	36.5	17.2
A388	21.9	22.6	18.2
S370	26.9	14.7	20.7
N373	24.4	11.6	14.5
W315	11.1	22.3	9.6
I387	14.8	4.2	7.1
**K385**	16.3	1.8	4.8
**K369**	13.4	3.1	3.0
T392	10.1	0.8	7.5
N362	13.4	0.4	3.7
N378	10.6	0.3	0.3
**K380**	10.2	0.2	0.1

[a]Residues are sorted by decreasing average frequency. Lysines are highlighted in bold.
[b]The frequency was calculated as average over all the replicas (production only). Only residues with a frequency ≥ 10% for at least one chain are reported.

Supplementary Table 7. Average frequency of occurrence of Lcl-CTD/C4S hydrogen bonds during MD simulations.

Residue[a]	Chain A[b] (%)	Chain B[b] (%)	Chain C[b] (%)
S371	39.7	36.8	40.8
S390	11.7	27.8	10.1
**K391**	19.1	11.7	9.7
T392	15.9	1.9	11.8
N373	10.6	7.8	8.4
S370	12.0	4.2	4.9
N362	14.6	0.7	4.6
**K385**	11.8	0.6	2.9

[a]Residues are sorted by decreasing average frequency. Lysines are highlighted in bold. Hydrogen bonding interactions were detected using Visual Molecular Dynamics[8] with a Donor-Acceptor distance threshold of 3.5 Å and a Hydrogen-Donor-Acceptor angle of 30°.
[b]The frequency was calculated as average over all the replicas (production only). When more than one donor/acceptor per residue was involved in hydrogen bonding, the frequency values were added together. Only residues with a frequency >= 10% for at least one chain are reported.

Supplementary References

1 Camilloni, C., De Simone, A., Vranken, W. F. & Vendruscolo, M. Determination of secondary structure populations in disordered states of proteins using nuclear magnetic resonance chemical shifts. *Biochemistry* **51**, 2224-2231, doi:10.1021/bi3001825 (2012).

2 Holm, L. & Sander, C. Dali: a network tool for protein structure comparison. *Trends Biochem Sci* **20**, 478-480, doi:10.1016/s0968-0004(00)89105-7 (1995).

3 Huang, K. F. *et al.* Crystal structure of a platelet-agglutinating factor isolated from the venom of Taiwan habu (Trimeresurus mucrosquamatus). *Biochem J* **378**, 399-407, doi:10.1042/BJ20031507 (2004).

4 Horii, K., Okuda, D., Morita, T. & Mizuno, H. Crystal structure of EMS16 in complex with the integrin alpha2-I domain. *J Mol Biol* **341**, 519-527, doi:10.1016/j.jmb.2004.06.036 (2004).

5 Luo, Y. *et al.* Crystal structure of enteropathogenic Escherichia coli intimin-receptor complex. *Nature* **405**, 1073-1077, doi:10.1038/35016618 (2000).

6 Hamburger, Z. A., Brown, M. S., Isberg, R. R. & Bjorkman, P. J. Crystal structure of invasin: a bacterial integrin-binding protein. *Science* **286**, 291-295 (1999).

7 Gautier, R., Douguet, D., Antonny, B. & Drin, G. HELIQUEST: a web server to screen sequences with specific alpha-helical properties. *Bioinformatics* **24**, 2101-2102, doi:10.1093/bioinformatics/btn392 (2008).

8 Humphrey, W., Dalke, A. & Schulten, K. VMD: visual molecular dynamics. *J Mol Graph* **14**, 33-38, 27-38, doi:10.1016/0263-7855(96)00018-5 (1996).
