# Supplementary material for: The Legionella collagen-like protein employs a distinct binding mechanism for the recognition of host glycosaminoglycans
Source: Nat Commun. 2024 Jun 8;15:4912. doi: 10.1038/s41467-024-49255-4 (PMC11162425; doi:10.1038/s41467-024-49255-4)
Supplement: Supplementary file 3 — Description of Additional Supplementary Files [file 41467_2024_49255_MOESM3_ESM.pdf]

Description of Additional Supplementary Files

File Name: Supplementary Data 1
Description: Lcl homologs from the DUF1566 gly_rich_SclB superfamily

File Name: Supplementary Data 2
Description: Bacterial strains used in this study

File Name: Supplementary Data 3
Description: Primers used in this study

File Name: Supplementary Data 4
Description: Plasmids used in this study

File Name: Supplementary Data 5
Description: Synthetic gene sequences
