# Supplementary material for: The Legionella collagen-like protein employs a distinct binding mechanism for the recognition of host glycosaminoglycans
Source: Nat Commun. 2024 Jun 8;15:4912. doi: 10.1038/s41467-024-49255-4 (PMC11162425; doi:10.1038/s41467-024-49255-4)
Supplement: Supplementary file 9 — Reporting Summary [file 41467_2024_49255_MOESM9_ESM.pdf]

Corresponding author(s): Arianna Fornili
Nicholas P. Cianciotto
James A. Garnett

Last updated by author(s): 2024-05-17

Reporting Summary

Nature Portfolio wishes to improve the reproducibility of the work that we publish. This form provides structure for consistency and transparency in reporting. For further information on Nature Portfolio policies, see our Editorial Policies and the Editorial Policy Checklist.

Statistics

For all statistical analyses, confirm that the following items are present in the figure legend, table legend, main text, or Methods section.

n/a	Confirmed	
☐	☒	The exact sample size (*n*) for each experimental group/condition, given as a discrete number and unit of measurement
☐	☒	A statement on whether measurements were taken from distinct samples or whether the same sample was measured repeatedly
☐	☒	The statistical test(s) used AND whether they are one- or two-sided<br>*Only common tests should be described solely by name; describe more complex techniques in the Methods section.*
☒	☐	A description of all covariates tested
☒	☐	A description of any assumptions or corrections, such as tests of normality and adjustment for multiple comparisons
☐	☒	A full description of the statistical parameters including central tendency (e.g. means) or other basic estimates (e.g. regression coefficient) AND variation (e.g. standard deviation) or associated estimates of uncertainty (e.g. confidence intervals)
☐	☒	For null hypothesis testing, the test statistic (e.g. *F*, *t*, *r*) with confidence intervals, effect sizes, degrees of freedom and *P* value noted<br>*Give P values as exact values whenever suitable.*
☒	☐	For Bayesian analysis, information on the choice of priors and Markov chain Monte Carlo settings
☒	☐	For hierarchical and complex designs, identification of the appropriate level for tests and full reporting of outcomes
☒	☐	Estimates of effect sizes (e.g. Cohen's *d*, Pearson's *r*), indicating how they were calculated

Our web collection on statistics for biologists contains articles on many of the points above.

Software and code

Policy information about availability of computer code

Data collection	Astra V, GROMACS 2019, TOPSPIN 3.6,
Data analysis	Astra V, CCP4, ATSAS, NMRPIPE, CCPNMR V2 and V3, MDanalysis, CHARMM

For manuscripts utilizing custom algorithms or software that are central to the research but not yet described in published literature, software must be made available to editors and reviewers. We strongly encourage code deposition in a community repository (e.g. GitHub). See the Nature Portfolio guidelines for submitting code & software for further information.

Data

Policy information about availability of data

All manuscripts must include a data availability statement. This statement should provide the following information, where applicable:

- Accession codes, unique identifiers, or web links for publicly available datasets
- A description of any restrictions on data availability
- For clinical datasets or third party data, please ensure that the statement adheres to our policy

Atomic coordinates and structure factors files generated in this study have been deposited in the Protein Data Bank database under accession codes 8Q4E (Lcl-CTD) and 8QK8 (Lcl-CTD/SO4). NMR assignments have been deposited in the Biological Magnetic Resonance Data Bank database under accession codes 52394 (Lcl-CTD) and 52395 (Lcl-CLR peptide). SAXS curves have been deposited in the Small Angle Scattering Data Bank database under accession codes SASDUG7 (Lcl-CTD WT), SASDUH7 (Lcl-CTD R477A trimer), SASDUJ7 (Lcl-CTD R477A monomer), SASDUK7 (Lcl-CTD E503A), SASDUL7 (Lcl-CTD K504A), SASDUM7 (Lcl-CTD K515A), SASDUN7 (Lcl-CTD K520A), SASDUP7 (Lcl-CTD D521A), SASDUQ7 (Lcl-CTD K526A). Initial and final structures from MD simulations are available at https://zenodo.org/doi/10.5281/zenodo.10961237 and https://zenodo.org/doi/10.5281/zenodo.10974841. The authors will provide raw data, additional information, and materials, including plasmids for protein expression, upon request, and should be addressed to J.G.

Research involving human participants, their data, or biological material

Policy information about studies with human participants or human data. See also policy information about sex, gender (identity/presentation), and sexual orientation and race, ethnicity and racism.

Reporting on sex and gender	N/A
Reporting on race, ethnicity, or other socially relevant groupings	N/A
Population characteristics	N/A
Recruitment	N/A
Ethics oversight	N/A

Note that full information on the approval of the study protocol must also be provided in the manuscript.

Field-specific reporting

Please select the one below that is the best fit for your research. If you are not sure, read the appropriate sections before making your selection.

☒ Life sciences ☐ Behavioural & social sciences ☐ Ecological, evolutionary & environmental sciences

For a reference copy of the document with all sections, see nature.com/documents/nr-reporting-summary-flat.pdf

Life sciences study design

All studies must disclose on these points even when the disclosure is negative.

Sample size	no sample size calc perfromed. min n=3 for student T-test
Data exclusions	no data exclusion
Replication	Reproducibility determined by visual inspection of raw plots. All replication successful
Randomization	Randomisation not appropriate for the studies or related statistical analysis
Blinding	Randomisation not appropriate for the studies or related statistical analysis

Reporting for specific materials, systems and methods

We require information from authors about some types of materials, experimental systems and methods used in many studies. Here, indicate whether each material, system or method listed is relevant to your study. If you are not sure if a list item applies to your research, read the appropriate section before selecting a response.

Materials & experimental systems

n/a	Involved in the study	
☐	☒	Antibodies
☒	☐	Eukaryotic cell lines
☒	☐	Palaeontology and archaeology
☒	☐	Animals and other organisms
☒	☐	Clinical data
☒	☐	Dual use research of concern
☒	☐	Plants

Methods

n/a	Involved in the study	
☒	☐	ChIP-seq
☒	☐	Flow cytometry
☒	☐	MRI-based neuroimaging

Antibodies

Antibodies used	anti-Lcl (Lampire Biological Laboratories, Pipersville, PA); goat anti-rabbit horseradish peroxidase antibody (Cell Signaling
Validation	Technology, Catalog #704); anti-His-HRP antibody (1 mg/ml; ThermoFisher Scientific, Catalog # MA1-21315-HRP)

Lcl antibody: validated by reacting with purified proteins and bacterial lysates on Western blot/immunoblotting
